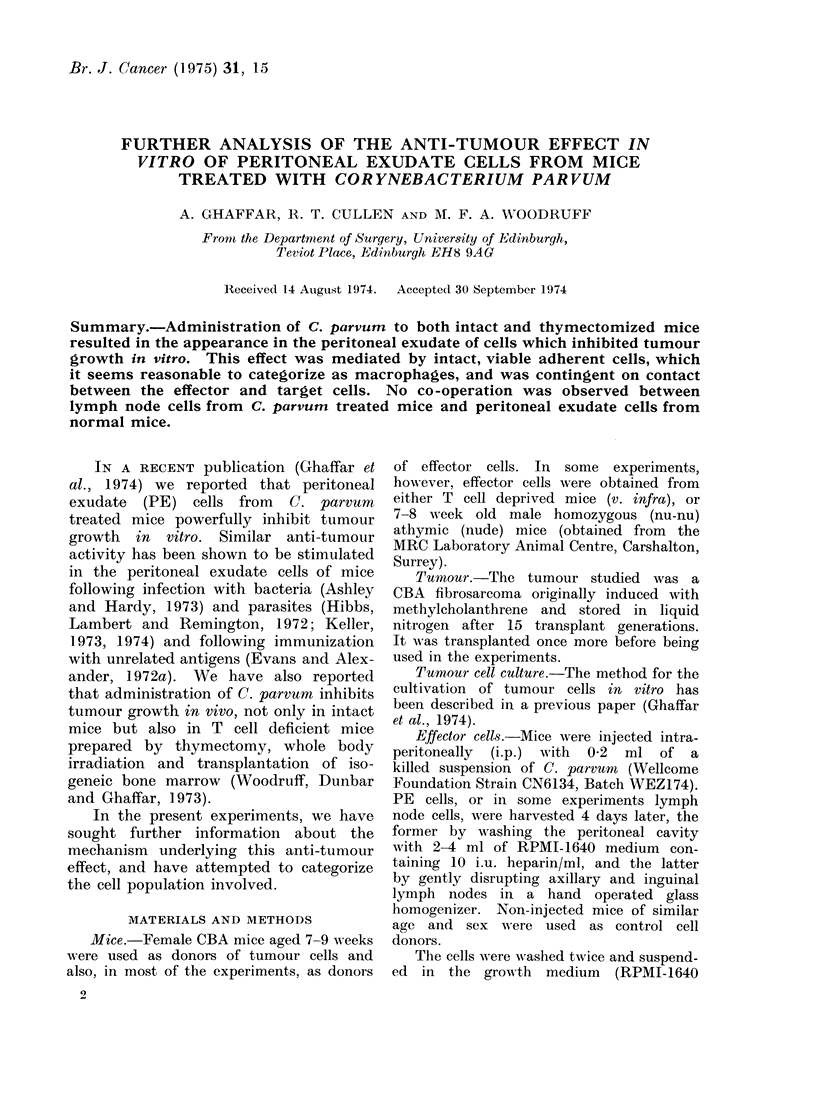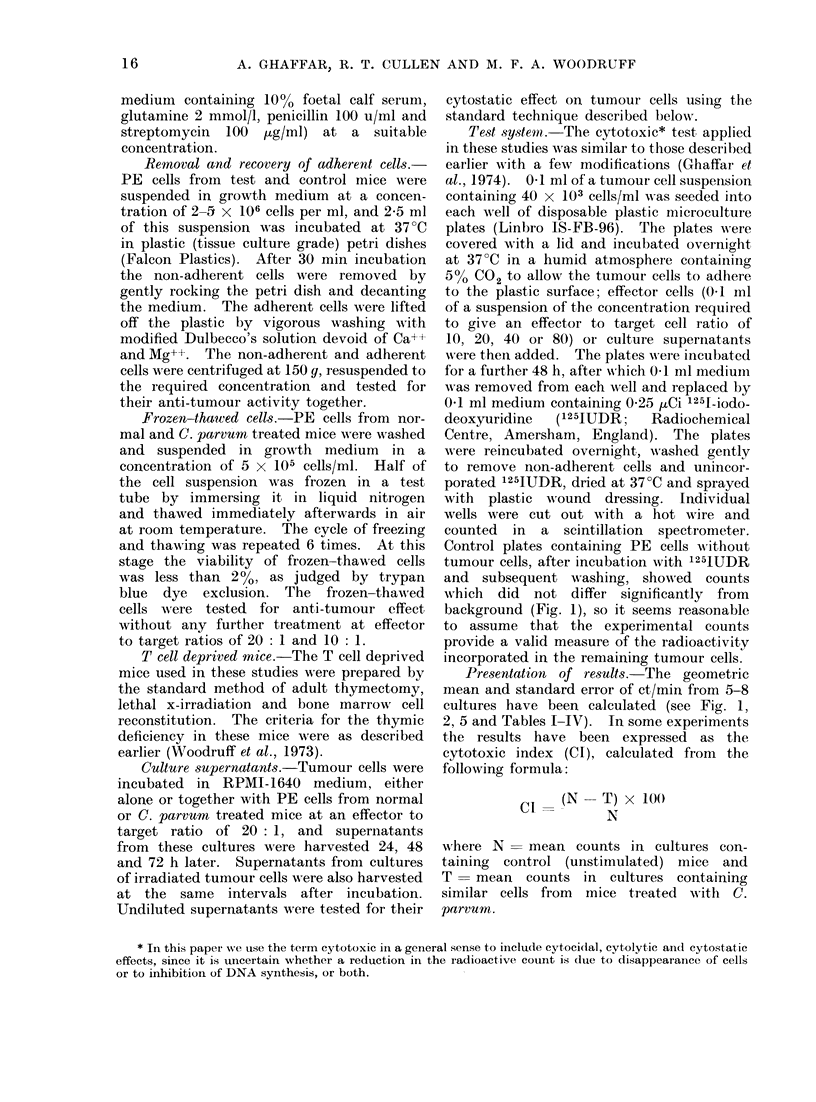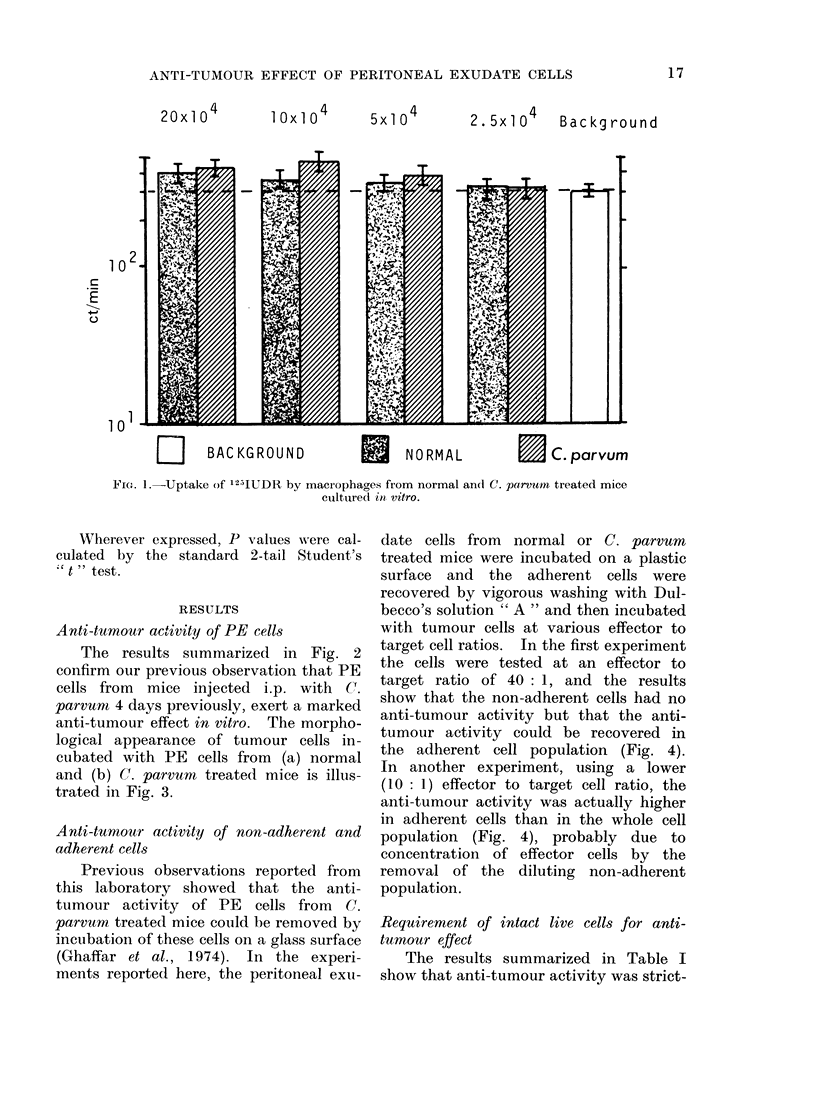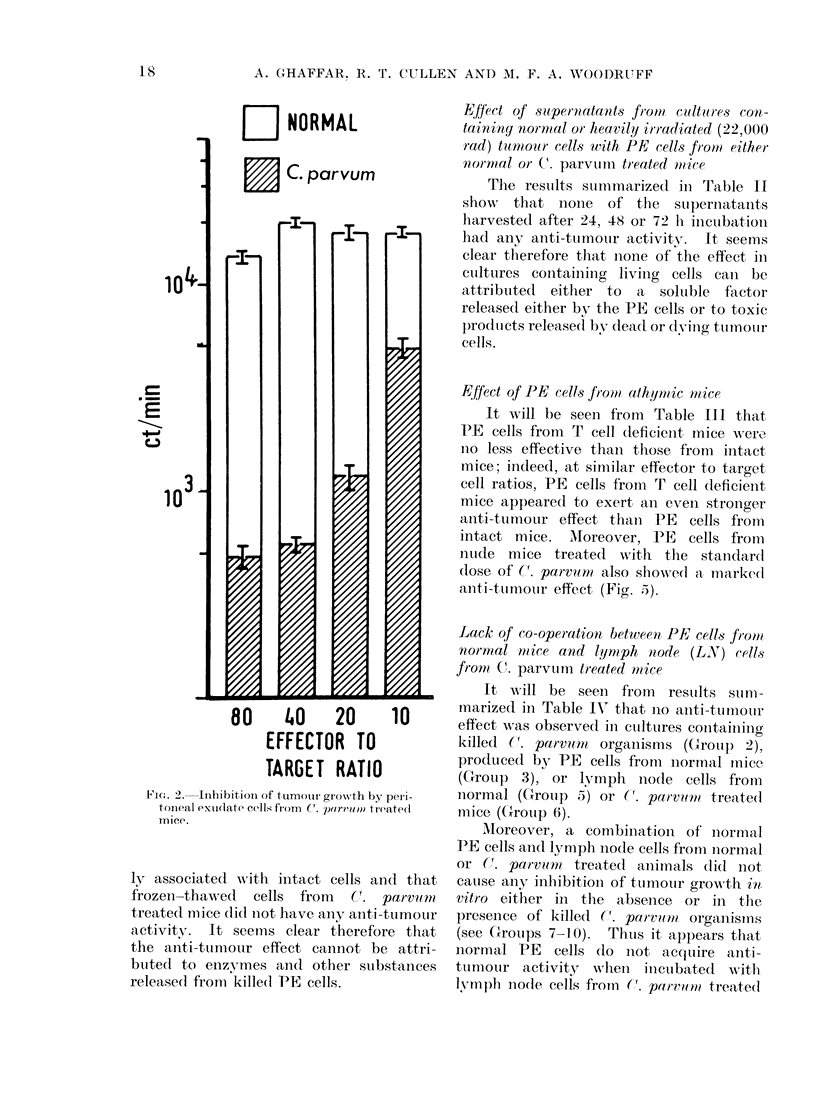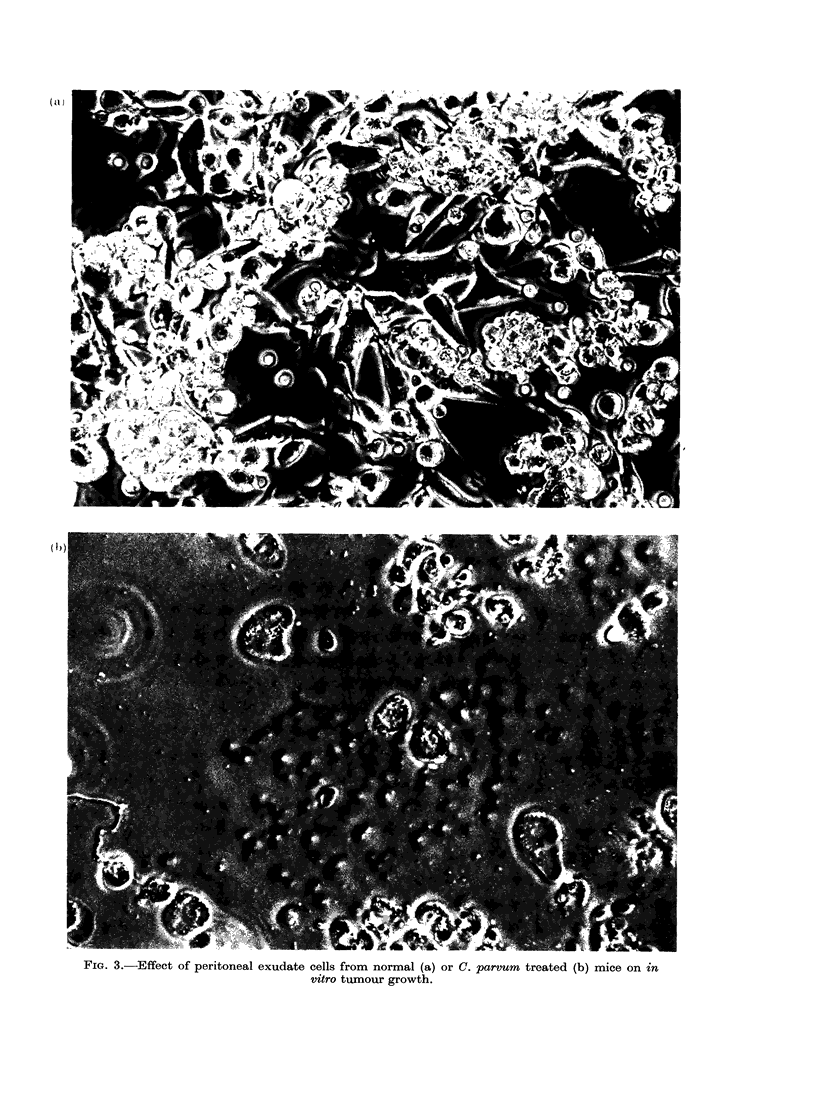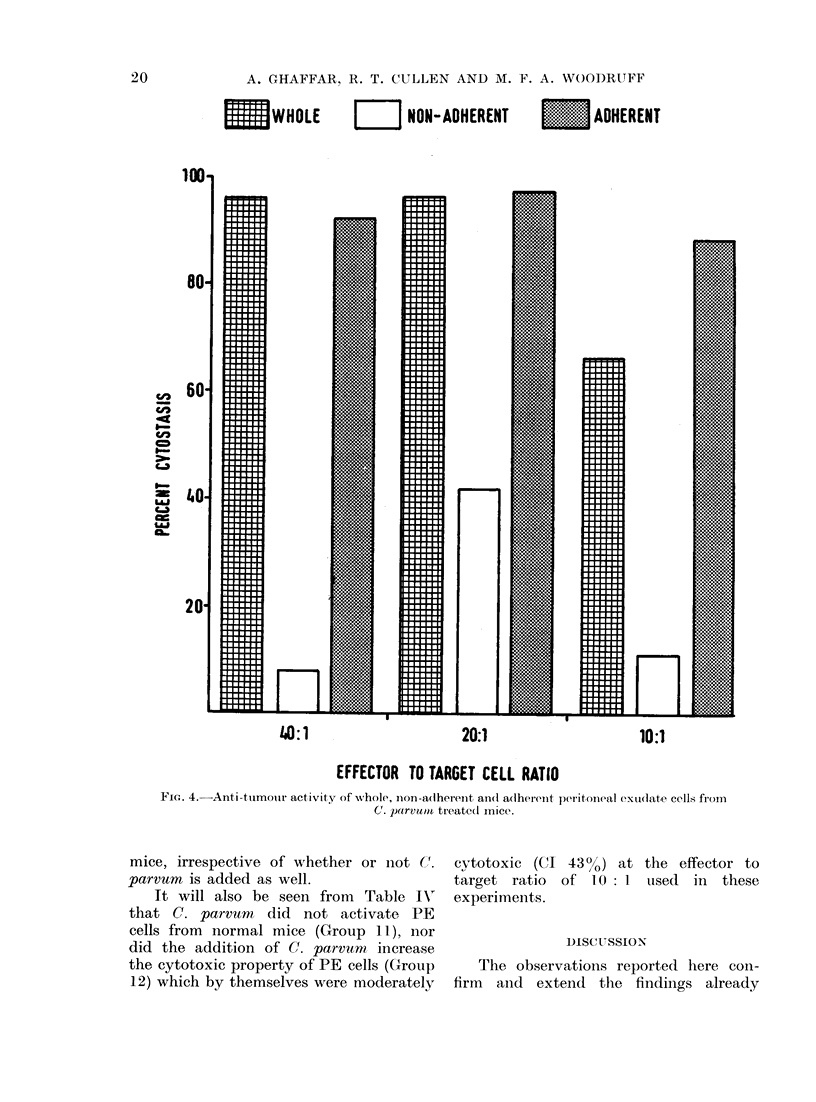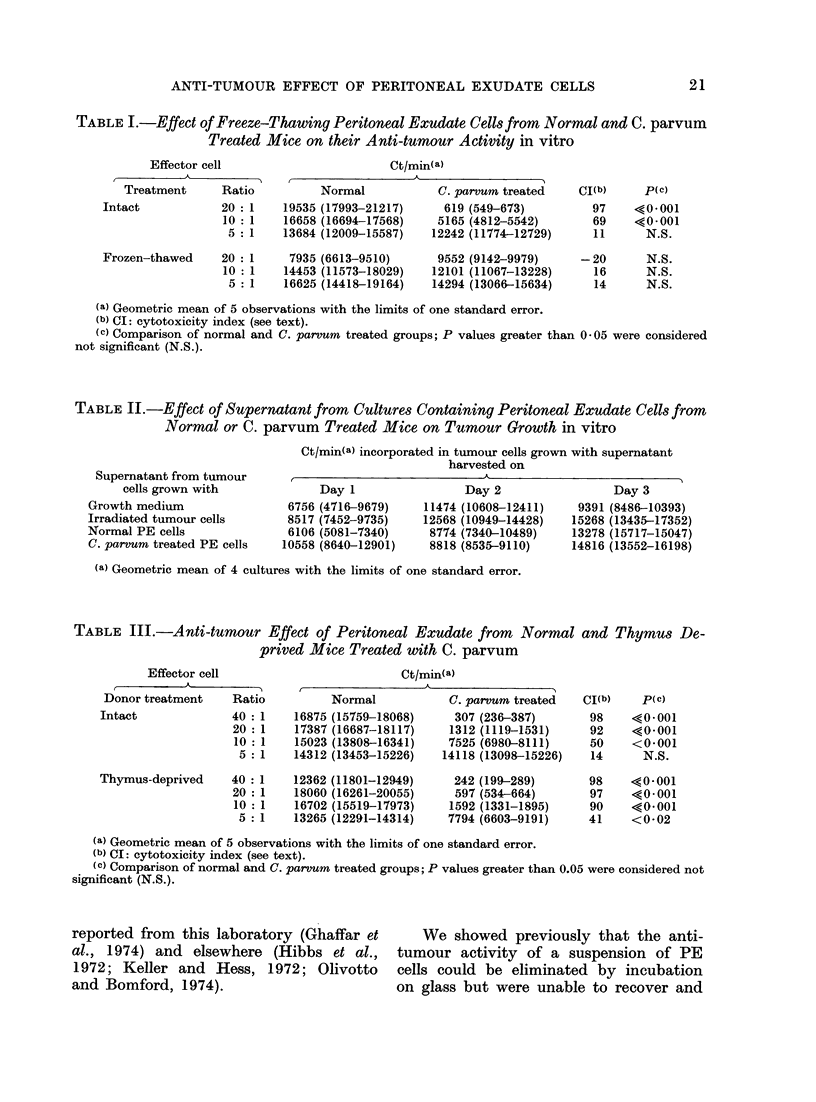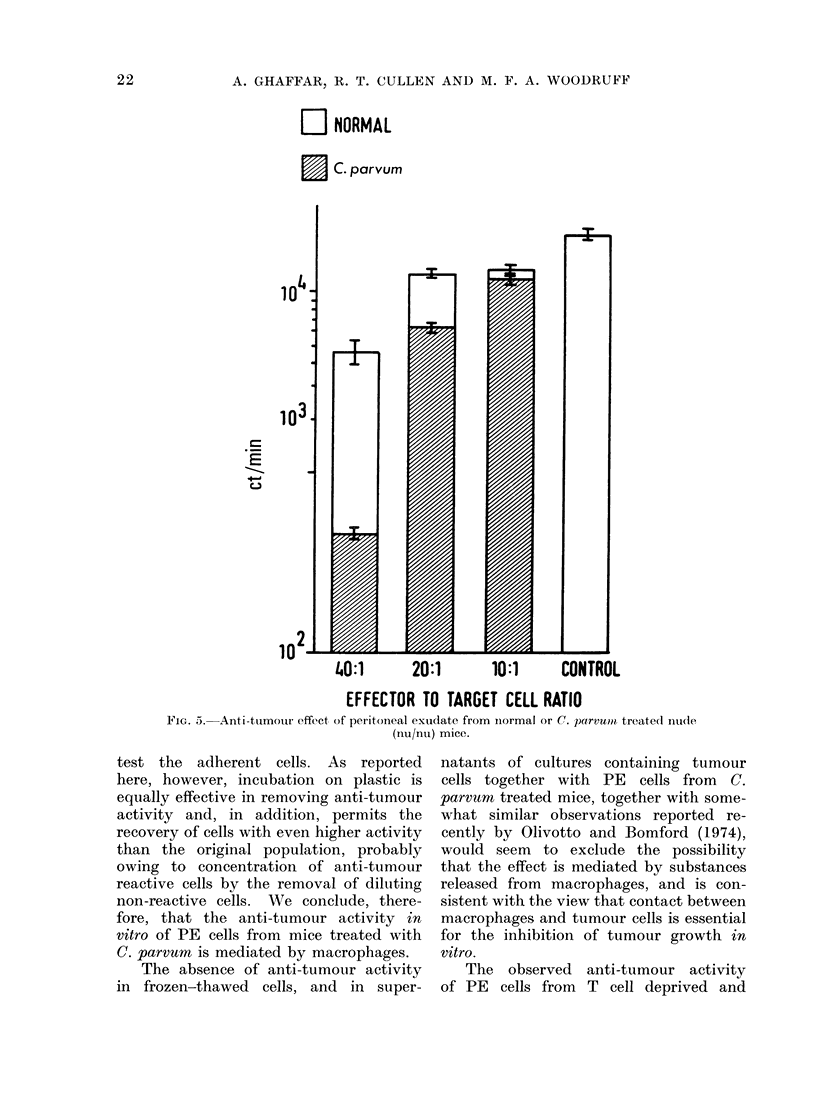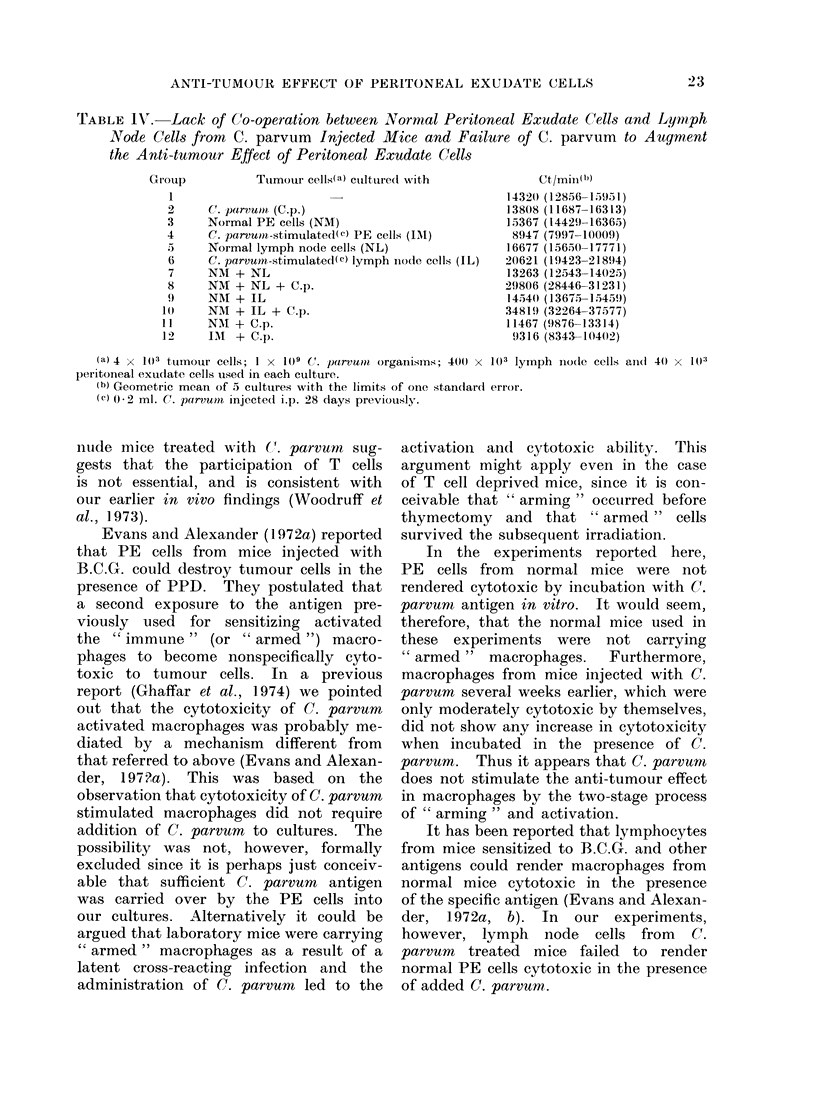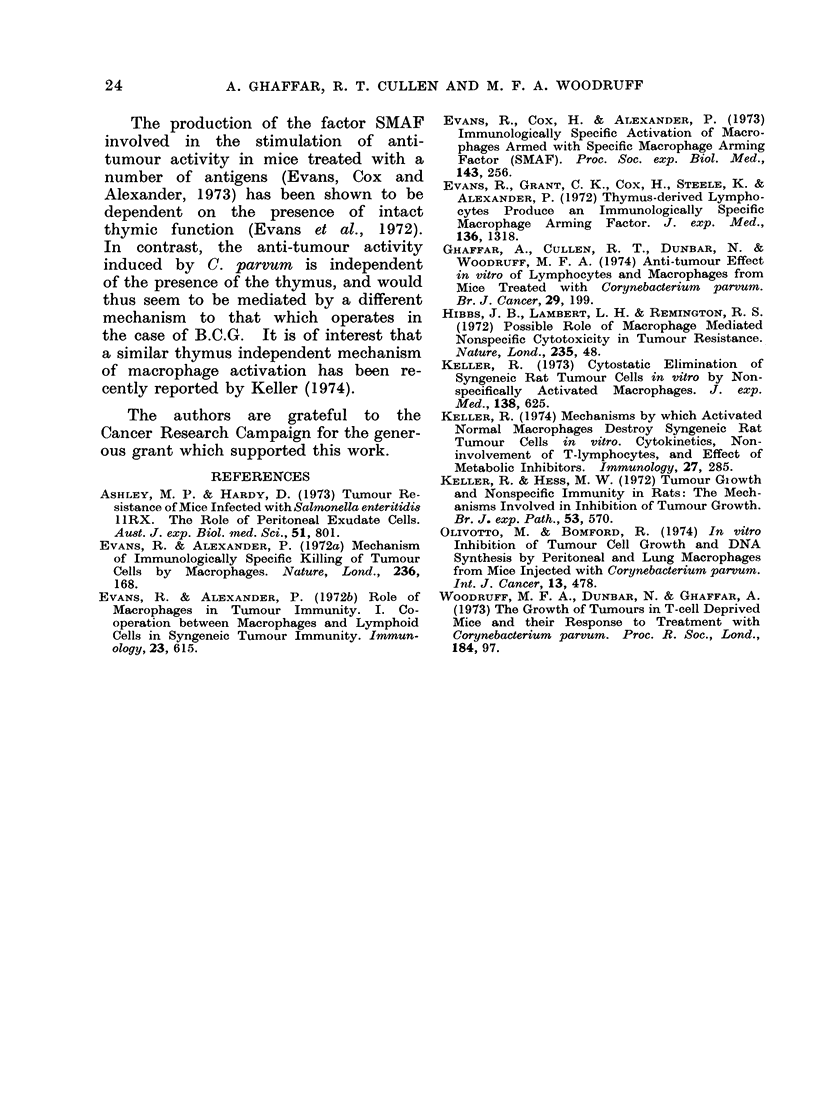# Further analysis of the anti-tumour effect in vitro of peritoneal exudate cells from mice treated with Corynebacterium parvum.

**DOI:** 10.1038/bjc.1975.3

**Published:** 1975-01

**Authors:** A. Ghaffar, R. T. Cullen, M. A. Woodruff

## Abstract

**Images:**


*Br. J. Cancer* (1975) **31**, 15

### FURTHER ANALYSIS OF THE ANTI-TUMOUR EFFECT *IN VITRO* OF PERITONEAL EXUDATE CELLS FROM MICE TREATED WITH *CORYNEBACTERIUM PARVUM*

A. GHAFFAR, R. T. CULLEN AND M. F. A. WOODRUFF

*From the Department of Surgery, University of Edinburgh, Teviot Place, Edinburgh EH8 9AG*

Received 14 August 1974. Accepted 30 September 1974

**Summary.—Administration of *C. parvum* to both intact and thymectomized mice resulted in the appearance in the peritoneal exudate of cells which inhibited tumour growth *in vitro*. This effect was mediated by intact, viable adherent cells, which it seems reasonable to categorize as macrophages, and was contingent on contact between the effector and target cells. No co-operation was observed between lymph node cells from *C. parvum* treated mice and peritoneal exudate cells from normal mice.**

IN A RECENT publication (Ghaffar *et al.*, 1974) we reported that peritoneal exudate (PE) cells from *C. parvum* treated mice powerfully inhibit tumour growth *in vitro*. Similar anti-tumour activity has been shown to be stimulated in the peritoneal exudate cells of mice following infection with bacteria (Ashley and Hardy, 1973) and parasites (Hibbs, Lambert and Remington, 1972; Keller, 1973, 1974) and following immunization with unrelated antigens (Evans and Alexander, 1972*a*). We have also reported that administration of *C. parvum* inhibits tumour growth *in vivo*, not only in intact mice but also in T cell deficient mice prepared by thymectomy, whole body irradiation and transplantation of isogeneic bone marrow (Woodruff, Dunbar and Ghaffar, 1973).

In the present experiments, we have sought further information about the mechanism underlying this anti-tumour effect, and have attempted to categorize the cell population involved.

### MATERIALS AND METHODS

*Mice.*—Female CBA mice aged 7–9 weeks were used as donors of tumour cells and also, in most of the experiments, as donors of effector cells. In some experiments, however, effector cells were obtained from either T cell deprived mice (*v. infra*), or 7–8 week old male homozygous (nu-nu) athymic (nude) mice (obtained from the MRC Laboratory Animal Centre, Carshalton, Surrey).

*Tumour.*—The tumour studied was a CBA fibrosarcoma originally induced with methylcholanthrene and stored in liquid nitrogen after 15 transplant generations. It was transplanted once more before being used in the experiments.

*Tumour cell culture.*—The method for the cultivation of tumour cells *in vitro* has been described in a previous paper (Ghaffar *et al.*, 1974).

*Effector cells.*—Mice were injected intraperitoneally (i.p.) with 0·2 ml of a killed suspension of *C. parvum* (Wellcome Foundation Strain CN6134, Batch WEZ174). PE cells, or in some experiments lymph node cells, were harvested 4 days later, the former by washing the peritoneal cavity with 2–4 ml of RPMI-1640 medium containing 10 i.u. heparin/ml, and the latter by gently disrupting axillary and inguinal lymph nodes in a hand operated glass homogenizer. Non-injected mice of similar age and sex were used as control cell donors.

The cells were washed twice and suspended in the growth medium (RPMI-1640

medium containing 10% foetal calf serum, glutamine 2 mmol/l, penicillin 100 u/ml and streptomycin 100 $\mu$g/ml) at a suitable concentration.

*Removal and recovery of adherent cells.*—PE cells from test and control mice were suspended in growth medium at a concentration of 2–5 $\times$ $10^6$ cells per ml, and 2·5 ml of this suspension was incubated at 37°C in plastic (tissue culture grade) petri dishes (Falcon Plastics). After 30 min incubation the non-adherent cells were removed by gently rocking the petri dish and decanting the medium. The adherent cells were lifted off the plastic by vigorous washing with modified Dulbecco's solution devoid of $Ca^{++}$ and $Mg^{++}$. The non-adherent and adherent cells were centrifuged at 150 *g*, resuspended to the required concentration and tested for their anti-tumour activity together.

*Frozen–thawed cells.*—PE cells from normal and *C. parvum* treated mice were washed and suspended in growth medium in a concentration of 5 $\times$ $10^5$ cells/ml. Half of the cell suspension was frozen in a test tube by immersing it in liquid nitrogen and thawed immediately afterwards in air at room temperature. The cycle of freezing and thawing was repeated 6 times. At this stage the viability of frozen–thawed cells was less than 2%, as judged by trypan blue dye exclusion. The frozen–thawed cells were tested for anti-tumour effect without any further treatment at effector to target ratios of 20 : 1 and 10 : 1.

*T cell deprived mice.*—The T cell deprived mice used in these studies were prepared by the standard method of adult thymectomy, lethal x-irradiation and bone marrow cell reconstitution. The criteria for the thymic deficiency in these mice were as described earlier (Woodruff *et al.*, 1973).

*Culture supernatants.*—Tumour cells were incubated in RPMI-1640 medium, either alone or together with PE cells from normal or *C. parvum* treated mice at an effector to target ratio of 20 : 1, and supernatants from these cultures were harvested 24, 48 and 72 h later. Supernatants from cultures of irradiated tumour cells were also harvested at the same intervals after incubation. Undiluted supernatants were tested for their cytostatic effect on tumour cells using the standard technique described below.

*Test system.*—The cytotoxic* test applied in these studies was similar to those described earlier with a few modifications (Ghaffar *et al.*, 1974). 0·1 ml of a tumour cell suspension containing 40 $\times$ $10^3$ cells/ml was seeded into each well of disposable plastic microculture plates (Linbro IS-FB-96). The plates were covered with a lid and incubated overnight at 37°C in a humid atmosphere containing 5% $CO_2$ to allow the tumour cells to adhere to the plastic surface; effector cells (0·1 ml of a suspension of the concentration required to give an effector to target cell ratio of 10, 20, 40 or 80) or culture supernatants were then added. The plates were incubated for a further 48 h, after which 0·1 ml medium was removed from each well and replaced by 0·1 ml medium containing 0·25 $\mu$Ci $^{125}$I-iododeoxyuridine ($^{125}$IUDR; Radiochemical Centre, Amersham, England). The plates were reincubated overnight, washed gently to remove non-adherent cells and unincorporated $^{125}$IUDR, dried at 37°C and sprayed with plastic wound dressing. Individual wells were cut out with a hot wire and counted in a scintillation spectrometer. Control plates containing PE cells without tumour cells, after incubation with $^{125}$IUDR and subsequent washing, showed counts which did not differ significantly from background (Fig. 1), so it seems reasonable to assume that the experimental counts provide a valid measure of the radioactivity incorporated in the remaining tumour cells.

*Presentation of results.*—The geometric mean and standard error of ct/min from 5–8 cultures have been calculated (see Fig. 1, 2, 5 and Tables I–IV). In some experiments the results have been expressed as the cytotoxic index (CI), calculated from the following formula:

$$CI = \frac{(N - T) \times 100}{N}$$

where N = mean counts in cultures containing control (unstimulated) mice and T = mean counts in cultures containing similar cells from mice treated with *C. parvum*.

* In this paper we use the term cytotoxic in a general sense to include cytocidal, cytolytic and cytostatic effects, since it is uncertain whether a reduction in the radioactive count is due to disappearance of cells or to inhibition of DNA synthesis, or both.

FIG. 1.—Uptake of $^{125}$IUDR by macrophages from normal and *C. parvum* treated mice cultured *in vitro*.

Wherever expressed, *P* values were calculated by the standard 2-tail Student's "*t*" test.

### RESULTS

### *Anti-tumour activity of PE cells*

The results summarized in Fig. 2 confirm our previous observation that PE cells from mice injected i.p. with *C. parvum* 4 days previously, exert a marked anti-tumour effect *in vitro*. The morphological appearance of tumour cells incubated with PE cells from (a) normal and (b) *C. parvum* treated mice is illustrated in Fig. 3.

### *Anti-tumour activity of non-adherent and adherent cells*

Previous observations reported from this laboratory showed that the anti-tumour activity of PE cells from *C. parvum* treated mice could be removed by incubation of these cells on a glass surface (Ghaffar *et al.*, 1974). In the experiments reported here, the peritoneal exudate cells from normal or *C. parvum* treated mice were incubated on a plastic surface and the adherent cells were recovered by vigorous washing with Dulbecco's solution " A " and then incubated with tumour cells at various effector to target cell ratios. In the first experiment the cells were tested at an effector to target ratio of 40 : 1, and the results show that the non-adherent cells had no anti-tumour activity but that the anti-tumour activity could be recovered in the adherent cell population (Fig. 4). In another experiment, using a lower (10 : 1) effector to target cell ratio, the anti-tumour activity was actually higher in adherent cells than in the whole cell population (Fig. 4), probably due to concentration of effector cells by the removal of the diluting non-adherent population.

### *Requirement of intact live cells for anti-tumour effect*

The results summarized in Table I show that anti-tumour activity was strict-

FIG. 2.—Inhibition of tumour growth by peritoneal exudate cells from *C. parvum* treated mice.

ly associated with intact cells and that frozen–thawed cells from *C. parvum* treated mice did not have any anti-tumour activity. It seems clear therefore that the anti-tumour effect cannot be attributed to enzymes and other substances released from killed PE cells.

*Effect of supernatants from cultures containing normal or heavily irradiated* (22,000 *rad*) *tumour cells with PE cells from either normal or* C. parvum *treated mice*

The results summarized in Table II show that none of the supernatants harvested after 24, 48 or 72 h incubation had any anti-tumour activity. It seems clear therefore that none of the effect in cultures containing living cells can be attributed either to a soluble factor released either by the PE cells or to toxic products released by dead or dying tumour cells.

*Effect of PE cells from athymic mice*

It will be seen from Table III that PE cells from T cell deficient mice were no less effective than those from intact mice; indeed, at similar effector to target cell ratios, PE cells from T cell deficient mice appeared to exert an even stronger anti-tumour effect than PE cells from intact mice. Moreover, PE cells from nude mice treated with the standard dose of *C. parvum* also showed a marked anti-tumour effect (Fig. 5).

*Lack of co-operation between PE cells from normal mice and lymph node* (*LN*) *cells from* C. parvum *treated mice*

It will be seen from results summarized in Table IV that no anti-tumour effect was observed in cultures containing killed *C. parvum* organisms (Group 2), produced by PE cells from normal mice (Group 3), or lymph node cells from normal (Group 5) or *C. parvum* treated mice (Group 6).

Moreover, a combination of normal PE cells and lymph node cells from normal or *C. parvum* treated animals did not cause any inhibition of tumour growth *in vitro* either in the absence or in the presence of killed *C. parvum* organisms (see Groups 7–10). Thus it appears that normal PE cells do not acquire anti-tumour activity when incubated with lymph node cells from *C. parvum* treated

(a)

(b)

FIG. 3.—Effect of peritoneal exudate cells from normal (a) or *C. parvum* treated (b) mice on *in vitro* tumour growth.

FIG. 4.—Anti-tumour activity of whole, non-adherent and adherent peritoneal exudate cells from *C. parvum* treated mice.

mice, irrespective of whether or not *C. parvum* is added as well.

It will also be seen from Table IV that *C. parvum* did not activate PE cells from normal mice (Group 11), nor did the addition of *C. parvum* increase the cytotoxic property of PE cells (Group 12) which by themselves were moderately cytotoxic (CI 43%) at the effector to target ratio of 10 : 1 used in these experiments.

### DISCUSSION

The observations reported here confirm and extend the findings already

TABLE I.—*Effect of Freeze–Thawing Peritoneal Exudate Cells from Normal and* C. parvum *Treated Mice on their Anti-tumour Activity* in vitro

| Effector cell | | Ct/min[a] | | | |
|---|---|---|---|---|---|
| Treatment | Ratio | Normal | *C. parvum* treated | CI[b] | $P$[c] |
| Intact | 20 : 1 | 19535 (17993–21217) | 619 (549–673) | 97 | ⩽0·001 |
| | 10 : 1 | 16658 (16694–17568) | 5165 (4812–5542) | 69 | ⩽0·001 |
| | 5 : 1 | 13684 (12009–15587) | 12242 (11774–12729) | 11 | N.S. |
| Frozen–thawed | 20 : 1 | 7935 (6613–9510) | 9552 (9142–9979) | −20 | N.S. |
| | 10 : 1 | 14453 (11573–18029) | 12101 (11067–13228) | 16 | N.S. |
| | 5 : 1 | 16625 (14418–19164) | 14294 (13066–15634) | 14 | N.S. |

(a) Geometric mean of 5 observations with the limits of one standard error.
(b) CI: cytotoxicity index (see text).
(c) Comparison of normal and *C. parvum* treated groups; $P$ values greater than 0·05 were considered not significant (N.S.).

TABLE II.—*Effect of Supernatant from Cultures Containing Peritoneal Exudate Cells from Normal or* C. parvum *Treated Mice on Tumour Growth* in vitro

| Supernatant from tumour cells grown with | Ct/min[a] incorporated in tumour cells grown with supernatant harvested on | | |
|---|---|---|---|
| | Day 1 | Day 2 | Day 3 |
| Growth medium | 6756 (4716–9679) | 11474 (10608–12411) | 9391 (8486–10393) |
| Irradiated tumour cells | 8517 (7452–9735) | 12568 (10949–14428) | 15268 (13435–17352) |
| Normal PE cells | 6106 (5081–7340) | 8774 (7340–10489) | 13278 (15717–15047) |
| *C. parvum* treated PE cells | 10558 (8640–12901) | 8818 (8535–9110) | 14816 (13552–16198) |

(a) Geometric mean of 4 cultures with the limits of one standard error.

TABLE III.—*Anti-tumour Effect of Peritoneal Exudate from Normal and Thymus Deprived Mice Treated with* C. parvum

| Effector cell | | Ct/min[a] | | | |
|---|---|---|---|---|---|
| Donor treatment | Ratio | Normal | *C. parvum* treated | CI[b] | $P$[c] |
| Intact | 40 : 1 | 16875 (15759–18068) | 307 (236–387) | 98 | ⩽0·001 |
| | 20 : 1 | 17387 (16687–18117) | 1312 (1119–1531) | 92 | ⩽0·001 |
| | 10 : 1 | 15023 (13808–16341) | 7525 (6980–8111) | 50 | <0·001 |
| | 5 : 1 | 14312 (13453–15226) | 14118 (13098–15226) | 14 | N.S. |
| Thymus-deprived | 40 : 1 | 12362 (11801–12949) | 242 (199–289) | 98 | ⩽0·001 |
| | 20 : 1 | 18060 (16261–20055) | 597 (534–664) | 97 | ⩽0·001 |
| | 10 : 1 | 16702 (15519–17973) | 1592 (1331–1895) | 90 | ⩽0·001 |
| | 5 : 1 | 13265 (12291–14314) | 7794 (6603–9191) | 41 | <0·02 |

(a) Geometric mean of 5 observations with the limits of one standard error.
(b) CI: cytotoxicity index (see text).
(c) Comparison of normal and *C. parvum* treated groups; $P$ values greater than 0.05 were considered not significant (N.S.).

reported from this laboratory (Ghaffar *et al.*, 1974) and elsewhere (Hibbs *et al.*, 1972; Keller and Hess, 1972; Olivotto and Bomford, 1974).

We showed previously that the anti-tumour activity of a suspension of PE cells could be eliminated by incubation on glass but were unable to recover and

FIG. 5.—Anti-tumour effect of peritoneal exudate from normal or *C. parvum* treated nude (nu/nu) mice.

test the adherent cells. As reported here, however, incubation on plastic is equally effective in removing anti-tumour activity and, in addition, permits the recovery of cells with even higher activity than the original population, probably owing to concentration of anti-tumour reactive cells by the removal of diluting non-reactive cells. We conclude, therefore, that the anti-tumour activity *in vitro* of PE cells from mice treated with *C. parvum* is mediated by macrophages.

The absence of anti-tumour activity in frozen–thawed cells, and in supernatants of cultures containing tumour cells together with PE cells from *C. parvum* treated mice, together with somewhat similar observations reported recently by Olivotto and Bomford (1974), would seem to exclude the possibility that the effect is mediated by substances released from macrophages, and is consistent with the view that contact between macrophages and tumour cells is essential for the inhibition of tumour growth *in vitro*.

The observed anti-tumour activity of PE cells from T cell deprived and

TABLE IV.—*Lack of Co-operation between Normal Peritoneal Exudate Cells and Lymph Node Cells from* C. parvum *Injected Mice and Failure of* C. parvum *to Augment the Anti-tumour Effect of Peritoneal Exudate Cells*

| Group | Tumour cells[(a)] cultured with | Ct/min[(b)] |
|---|---|---|
| 1 | — | 14320 (12856–15951) |
| 2 | *C. parvum* (C.p.) | 13808 (11687–16313) |
| 3 | Normal PE cells (NM) | 15367 (14429–16365) |
| 4 | *C. parvum*-stimulated[(c)] PE cells (IM) | 8947 (7997–10009) |
| 5 | Normal lymph node cells (NL) | 16677 (15650–17771) |
| 6 | *C. parvum*-stimulated[(c)] lymph node cells (IL) | 20621 (19423–21894) |
| 7 | NM + NL | 13263 (12543–14025) |
| 8 | NM + NL + C.p. | 29806 (28446–31231) |
| 9 | NM + IL | 14540 (13675–15459) |
| 10 | NM + IL + C.p. | 34819 (32264–37577) |
| 11 | NM + C.p. | 11467 (9876–13314) |
| 12 | IM + C.p. | 9316 (8343–10402) |

(a) $4 \times 10^3$ tumour cells; $1 \times 10^9$ *C. parvum* organisms; $400 \times 10^3$ lymph node cells and $40 \times 10^3$ peritoneal exudate cells used in each culture.

(b) Geometric mean of 5 cultures with the limits of one standard error.

(c) 0·2 ml. *C. parvum* injected i.p. 28 days previously.

nude mice treated with *C. parvum* suggests that the participation of T cells is not essential, and is consistent with our earlier *in vivo* findings (Woodruff *et al.*, 1973).

Evans and Alexander (1972*a*) reported that PE cells from mice injected with B.C.G. could destroy tumour cells in the presence of PPD. They postulated that a second exposure to the antigen previously used for sensitizing activated the "immune" (or "armed") macrophages to become nonspecifically cytotoxic to tumour cells. In a previous report (Ghaffar *et al.*, 1974) we pointed out that the cytotoxicity of *C. parvum* activated macrophages was probably mediated by a mechanism different from that referred to above (Evans and Alexander, 197?*a*). This was based on the observation that cytotoxicity of *C. parvum* stimulated macrophages did not require addition of *C. parvum* to cultures. The possibility was not, however, formally excluded since it is perhaps just conceivable that sufficient *C. parvum* antigen was carried over by the PE cells into our cultures. Alternatively it could be argued that laboratory mice were carrying "armed" macrophages as a result of a latent cross-reacting infection and the administration of *C. parvum* led to the activation and cytotoxic ability. This argument might apply even in the case of T cell deprived mice, since it is conceivable that "arming" occurred before thymectomy and that "armed" cells survived the subsequent irradiation.

In the experiments reported here, PE cells from normal mice were not rendered cytotoxic by incubation with *C. parvum* antigen *in vitro*. It would seem, therefore, that the normal mice used in these experiments were not carrying "armed" macrophages. Furthermore, macrophages from mice injected with *C. parvum* several weeks earlier, which were only moderately cytotoxic by themselves, did not show any increase in cytotoxicity when incubated in the presence of *C. parvum*. Thus it appears that *C. parvum* does not stimulate the anti-tumour effect in macrophages by the two-stage process of "arming" and activation.

It has been reported that lymphocytes from mice sensitized to B.C.G. and other antigens could render macrophages from normal mice cytotoxic in the presence of the specific antigen (Evans and Alexander, 1972*a*, *b*). In our experiments, however, lymph node cells from *C. parvum* treated mice failed to render normal PE cells cytotoxic in the presence of added *C. parvum*.

The production of the factor SMAF involved in the stimulation of anti-tumour activity in mice treated with a number of antigens (Evans, Cox and Alexander, 1973) has been shown to be dependent on the presence of intact thymic function (Evans *et al.*, 1972). In contrast, the anti-tumour activity induced by *C. parvum* is independent of the presence of the thymus, and would thus seem to be mediated by a different mechanism to that which operates in the case of B.C.G. It is of interest that a similar thymus independent mechanism of macrophage activation has been recently reported by Keller (1974).

The authors are grateful to the Cancer Research Campaign for the generous grant which supported this work.

### REFERENCES

ASHLEY, M. P. & HARDY, D. (1973) Tumour Resistance of Mice Infected with *Salmonella enteritidis* 11RX. The Role of Peritoneal Exudate Cells. *Aust. J. exp. Biol. med. Sci.*, **51**, 801.

EVANS, R. & ALEXANDER, P. (1972*a*) Mechanism of Immunologically Specific Killing of Tumour Cells by Macrophages. *Nature, Lond.*, **236**, 168.

EVANS, R. & ALEXANDER, P. (1972*b*) Role of Macrophages in Tumour Immunity. I. Cooperation between Macrophages and Lymphoid Cells in Syngeneic Tumour Immunity. *Immunology*, **23**, 615.

EVANS, R., COX, H. & ALEXANDER, P. (1973) Immunologically Specific Activation of Macrophages Armed with Specific Macrophage Arming Factor (SMAF). *Proc. Soc. exp. Biol. Med.*, **143**, 256.

EVANS, R., GRANT, C. K., COX, H., STEELE, K. & ALEXANDER, P. (1972) Thymus-derived Lymphocytes Produce an Immunologically Specific Macrophage Arming Factor. *J. exp. Med.*, **136**, 1318.

GHAFFAR, A., CULLEN, R. T., DUNBAR, N. & WOODRUFF, M. F. A. (1974) Anti-tumour Effect *in vitro* of Lymphocytes and Macrophages from Mice Treated with *Corynebacterium parvum*. *Br. J. Cancer*, **29**, 199.

HIBBS, J. B., LAMBERT, L. H. & REMINGTON, R. S. (1972) Possible Role of Macrophage Mediated Nonspecific Cytotoxicity in Tumour Resistance. *Nature, Lond.*, **235**, 48.

KELLER, R. (1973) Cytostatic Elimination of Syngeneic Rat Tumour Cells *in vitro* by Nonspecifically Activated Macrophages. *J. exp. Med.*, **138**, 625.

KELLER, R. (1974) Mechanisms by which Activated Normal Macrophages Destroy Syngeneic Rat Tumour Cells *in vitro*. Cytokinetics, Noninvolvement of T-lymphocytes, and Effect of Metabolic Inhibitors. *Immunology*, **27**, 285.

KELLER, R. & HESS, M. W. (1972) Tumour Growth and Nonspecific Immunity in Rats: The Mechanisms Involved in Inhibition of Tumour Growth. *Br. J. exp. Path.*, **53**, 570.

OLIVOTTO, M. & BOMFORD, R. (1974) *In vitro* Inhibition of Tumour Cell Growth and DNA Synthesis by Peritoneal and Lung Macrophages from Mice Injected with *Corynebacterium parvum*. *Int. J. Cancer*, **13**, 478.

WOODRUFF, M. F. A., DUNBAR, N. & GHAFFAR, A. (1973) The Growth of Tumours in T-cell Deprived Mice and their Response to Treatment with *Corynebacterium parvum*. *Proc. R. Soc., Lond.*, **184**, 97.